# Spatial-Temporal Functional Mapping Combined With Cortico-Cortical Evoked Potentials in Predicting Cortical Stimulation Results

**DOI:** 10.3389/fnhum.2021.661976

**Published:** 2021-04-14

**Authors:** Yujing Wang, Mark A. Hays, Christopher Coogan, Joon Y. Kang, Adeen Flinker, Ravindra Arya, Anna Korzeniewska, Nathan E. Crone

**Affiliations:** ^1^Department of Neurology, Johns Hopkins University School of Medicine, Baltimore, MD, United States; ^2^Department of Biomedical Engineering, Johns Hopkins University School of Medicine, Baltimore, MD, United States; ^3^Department of Neurology, New York University School of Medicine, New York, NY, United States; ^4^Comprehensive Epilepsy Center, Division of Neurology, Cincinnati Children’s Hospital Medical Center, Cincinnati, OH, United States; ^5^Department of Pediatrics, University of Cincinnati College of Medicine, Cincinnati, OH, United States

**Keywords:** language functional mapping, electrocortical stimulation, high gamma activation, effective connectivity, cortico-cortical evoked potentials

## Abstract

Functional human brain mapping is commonly performed during invasive monitoring with intracranial electroencephalographic (iEEG) electrodes prior to resective surgery for drug­ resistant epilepsy. The current gold standard, electrocortical stimulation mapping (ESM), is time ­consuming, sometimes elicits pain, and often induces after discharges or seizures. Moreover, there is a risk of overestimating eloquent areas due to propagation of the effects of stimulation to a broader network of language cortex. Passive iEEG spatial-temporal functional mapping (STFM) has recently emerged as a potential alternative to ESM. However, investigators have observed less correspondence between STFM and ESM maps of language than between their maps of motor function. We hypothesized that incongruities between ESM and STFM of language function may arise due to propagation of the effects of ESM to cortical areas having strong effective connectivity with the site of stimulation. We evaluated five patients who underwent invasive monitoring for seizure localization, whose language areas were identified using ESM. All patients performed a battery of language tasks during passive iEEG recordings. To estimate the effective connectivity of stimulation sites with a broader network of task-activated cortical sites, we measured cortico-cortical evoked potentials (CCEPs) elicited across all recording sites by single-pulse electrical stimulation at sites where ESM was performed at other times. With the combination of high gamma power as well as CCEPs results, we trained a logistic regression model to predict ESM results at individual electrode pairs. The average accuracy of the classifier using both STFM and CCEPs results combined was 87.7%, significantly higher than the one using STFM alone (71.8%), indicating that the correspondence between STFM and ESM results is greater when effective connectivity between ESM stimulation sites and task-activated sites is taken into consideration. These findings, though based on a small number of subjects to date, provide preliminary support for the hypothesis that incongruities between ESM and STFM may arise in part from propagation of stimulation effects to a broader network of cortical language sites activated by language tasks, and suggest that more studies, with larger numbers of patients, are needed to understand the utility of both mapping techniques in clinical practice.

## Introduction

Despite ongoing advances in non-invasive functional neuroimaging, electrocortical stimulation mapping (ESM) remains the gold standard for mapping cortical function in individual patients at a sufficiently fine spatial scale to guide the surgical resection of brain tissue for the treatment of drug-resistant epilepsy (Penfield and Jasper, 1954; Lesser et al., 1994) and brain tumors (Sanai et al., 2008). The major advantage of this technique is that it allows clinicians to simulate the neurological consequences of lesioning tissue before it is permanently resected (Ojemann et al., 1989). However, there are important practical limitations on its clinical application. Chief amongst these is the risk of triggering afterdischarges and clinical seizures (Lesser et al., 1984; Blume et al., 2004; Hamberger, 2007; Aungaroon et al., 2017) that can prevent comprehensive functional mapping. Additionally, ESM can elicit pain that prevents mapping at individual sites (Lesser et al., 1985). Last, because ESM is done sequentially at pairs of electrodes, each time finding the optimal stimulation current (Lesser et al., 1984; Pouratian et al., 2004) and then testing the effect of stimulation on different language tasks (Schäffler et al., 1993), it is time-consuming, which often forces clinicians to map only a subset of sites. This factor may ultimately pose a particularly acute limitation on ESM as the number and density of intracranial electroencephalographic (iEEG) electrodes used for long-term monitoring increases (Viventi et al., 2011; Bouchard and Chang, 2014).

In addition to the practical limitations on ESM’s clinical application, there are concerns about its accuracy and predictive value. The neural populations and operations that are interrupted during stimulation are not well controlled, and it is difficult to rule out distant effects through diaschisis or the distant effects of action potentials evoked by stimulation (Ishitobi et al., 2000; Hamberger, 2007; Karakis et al., 2015). Furthermore, the simulated lesion of ESM cannot take into account the reorganization that occurs after real permanent lesions, and if it is done in only a subset of electrodes, it cannot identify other cortical sites that could potentially assume the function of the lesioned site (i.e., assess functional reserve). Finally, when ESM interrupts the performance of a cognitive task such as word production, the effect is usually all-or-none. The same observed effect can potentially result from interruption of different stages of processing or levels of representation that are necessary for successful task completion.

The limitations of ESM have long motivated the investigation of passive iEEG recordings as a tool for mapping cortical function prior to resective surgery (Crone et al., 1998a, 2001; Grossman and Gotman, 2001; Sinai et al., 2005; Cervenka et al., 2013). Intracranial EEG recordings, such as electrocorticography (ECoG) and stereo electroencephalographic (SEEG), cannot trigger seizures or pain, and they can be used to simultaneously survey task-related cortical activity in the entire set of implanted electrodes. Moreover, iEEG recordings yield a graded measure of task-related neural activity capable of resolving the activation of cortical sites at temporal scales comparable to the stages of processing that comprise language tasks (Edwards et al., 2010; Arya et al., 2019a). Thus, the relative degree and timing of activation at a given site can be used to estimate its contribution to these processing stages, providing clinicians with more detailed information as they weigh the benefits and risks of removing epileptogenic tissue vs. sparing eloquent cortex.

Despite the practical and theoretical advantages of iEEG spatial-temporal functional mapping (STFM) over ESM, STFM has not been widely used in clinical practice. One reason for this has been a lack of consensus on which signal components are most informative about task-related neural activity. In recent years, high gamma (60–200 Hz) power changes have been increasingly recognized as a robust and reliable index of task-related activation of cortical populations of neurons (Crone et al., 2006, 2011; Jerbi et al., 2009; Lachaux et al., 2012). This index is highly correlated with blood oxygen level-dependent (BOLD) responses in fMRI (Lachaux et al., 2007; Khursheed et al., 2011; Siero et al., 2014; Genetti et al., 2015) and with single unit activity recorded by microelectrodes (Ray et al., 2008; Manning et al., 2009). Accordingly, it is highly specific with respect to the location and timing of task-related cortical activation, and it has been observed in nearly every cortical functional-anatomical domain in which it has been studied, including sensorimotor, auditory, visual, and language areas (Jerbi et al., 2009; Crone et al., 2011; Lachaux et al., 2012). Moreover, recent technological developments have allowed STFM to be performed online, providing immediate feedback to clinicians (Wang et al., 2016; Milsap et al., 2019). STFM can also illuminate the temporal sequence of network activation (dynamics) with a time resolution that is far superior to fMRI, allowing greater insights into the functional role of individual sites.

These properties have made STFM an attractive tool for human research in cognitive and systems neuroscience. Indeed, recent studies have demonstrated extraordinary spatial and temporal selectivity in iEEG-recorded population responses (Flinker et al., 2010; Wang et al., 2021). In spite of these advances in research, the potential clinical application of STFM for pre-resective functional mapping has not yet been fully realized. To date, efforts to demonstrate the clinical utility of STFM have used ESM for validation, and although several studies have found strong agreement between iEEG high gamma responses and ESM in motor and early sensory cortices (Crone et al., 1998b; Miller et al., 2007; Brunner et al., 2009; Sinai et al., 2009), there has been less agreement in language cortex (Sinai et al., 2005; Towle et al., 2008; Wu et al., 2010; Miller et al., 2011; Bauer et al., 2013; Arya et al., 2017, 2018; Babajani-Feremi et al., 2018). For example, in a study comparing STFM and ESM for localization of object naming in 13 patients (Sinai et al., 2005), the authors observed a tradeoff between sensitivity and specificity as the threshold for the magnitude of high gamma responses was varied, i.e., low thresholds yielded high sensitivity but low specificity and *vice versa*.

The reliance of spoken word production on the function of large-scale cortical networks in frontal, parietal, and temporal lobes has long been appreciated by behavioral neurologists and cognitive psychologists studying the effects of lesions on different brain regions. These effects can be exquisitely specific for different aspects of language function, including perceptual processing, semantic and phonological representations, and articulatory plans. Psychophysical investigations into the timing of these different cognitive operations have indicated that they occur in quasi-sequential stages that overlap in time (cascaded) (Indefrey and Levelt, 2004). EEG, MEG, and fMRI studies have provisionally localized these operations to different brain regions, but because of variations in functional anatomy, these insights cannot be clinically applied to individual patients (Ojemann, 1979; Rademacher et al., 1993). In contrast, iEEG high gamma power changes are sufficiently robust to yield statistically significant responses within individuals, revealing the location and timing of cortical processing at clinically useful resolutions. However, processing at a given site may not always be critical to task performance. Furthermore, processing at each stage likely occurs in sub-networks comprised of multiple cortical sites. The opportunity, and challenge, for iEEG STFM is thus to identify which sites in these sub-networks are most important for task performance so that impairments can be avoided.

Evidence from a variety of sources has called into question the assumption that the effects of ESM are restricted to tissue underneath each stimulating electrode (Ishitobi et al., 2000; Matsumoto et al., 2004; Karakis et al., 2015). Based on these findings, we hypothesize that at least some false negative STFM sites are due to propagation of ESM to distant nodes in a broader network of task-activated sites, resulting in task interference. We evaluated five patients who underwent invasive monitoring for seizure localization whose language and motor areas were identified using ESM. Additionally, all patients performed language tasks including picture naming, auditory naming, word reading and auditory word repetition during passive iEEG recordings. We also measured cortico-cortical evoked potentials (CCEPs) elicited by single-pulse electrical stimulation at distant cortical sites when the patients were awake and at rest. With the combination of high gamma power and CCEP results, we trained a logistic regression model to predict ESM results at individual electrode pairs. Our findings suggest that additional information about effective connectivity in the overall network of cortical regions activated by language tasks can enhance the ability of iEEG STFM to predict ESM results.

## Materials and Methods

### Patient Information

Five English speaking patients (Table 1) with intractable epilepsy underwent placement of subdural electrodes in the left (dominant) hemisphere to localize their ictal onset zone and to identify language and motor areas using electrocortical stimulation mapping. In Patients 1–4, the implanted electrodes consisted of subdural arrays (grids and/or strips) of standard electrodes (2.3 mm exposed diameter, 1 cm center-to-center spacing, Adtech, Racine, WI, USA or PMT Crop, Chanhassen, MN, USA) as well as high-density electrodes (2 mm exposed diameter, 5 mm center-to-center spacing, PMT Crop, Chanhassen, MN, USA). In Patient 5, the implanted electrodes consisted of SEEG depth electrodes (0.86 mm diameter, 5–10 mm contact spacing, Adtech, Racine, WI, USA) implanted using a ROSA intraoperative robot (Zimmer Biomet Robotics, Montpellier, France). In all patients, the anatomical placement of electrodes was dictated solely by clinical considerations for recording seizures or mapping cortical function.

**Table 1:** Patient demographic and clinical information.

Patient	1	2	3	4	5
Age	25	32	26	49	42
Gender	M	M	F	M	F
Handedness	Right	Right	Both	Right	Right
Hemisphere Dominance	Left	Left	Left	Left	Left
Hemispheric coverage	Left	Left	Left	Left	Left
Seizure onset zone	Ventral left precentral gyrus + left inferior premotor area	Left superior parietal lobule	Left frontal lobe	Left fronto-central head regions	Bilateral neo-cortical temporal regions
Tasks performed	Word reading, Picture naming	Word reading, Word repetition	Word reading, Picture naming, Auditory naming	Word repetition, Picture naming, Auditory naming	Word reading, Word repetition, Picture naming

### Standard Protocol Approvals, Registrations, and Patient Consents

Patients were admitted to the Johns Hopkins Epilepsy Monitoring Unit after electrode implantation for a period of 6–14 days. All patients gave informed consent to participate in research testing under a protocol approved by the Institutional Review Board of the Johns Hopkins Medical Institutions.

### Experimental Paradigm

A battery of language tasks was performed under ESM and STFM (Shum et al., 2020). In the word reading task (STFM only), subjects were shown a word on a monitor directly in front of them and were instructed to read it out loud. In a paragraph reading task (ESM only), subjects read stories aloud as electrocortical stimulation was intermittently given. In the picture naming task (STFM and ESM), subjects were shown a picture of an object, as a stimulus on a monitor (STFM) or a piece of article (ESM) directly in front of them. Subjects were instructed to speak the name of the object in the picture or say “pass” if they could not recall the name (STFM only). In the auditory word repetition task (STFM only), subjects were played an audio recording of a spoken word through a speaker placed in front of them. Subjects were instructed to verbally repeat the cued word. In the auditory naming task (STFM and ESM), subjects were played an audio recording of a spoken sentence describing a certain object through a speaker placed in front of them. They were instructed to verbally name the object out loud. Trial numbers of each task for each patient were governed by the time constraints on patient testing and the set of stimuli used (50–100 trials for STFM, 15–30 trials for ESM).

### Electrode Localization

Electrode locations were identified in a high-resolution post-operative brain CT; they were then transformed onto a high-resolution pre-operative brain MRI by volumetrically co-registering the pre- and post-operative scans in Bioimage Suite (Duncan et al., 2004).

### Data Acquisition and Analysis

Recordings of all standard and high-density electrodes were referenced to a single intracranial electrode to minimize extracranial sources of artifact. Raw iEEG signals were recorded with a 256-channel recording system (NeuroPort, BlackRock Microsystems, Salt Lake City, UT, USA; or Nihon Kohden America, Irvine, CA, USA), which amplified and sampled the data at a minimum of 1 kHz and a maximum of 30 kHz of sampling rate with an analog third-order Butterworth anti-aliasing filter. The anti-aliased recording was decimated to 1 kHz in all patients prior to any subsequent analysis. Channels with excessive amounts of noise were excluded from analysis. To remove spatial bias in the raw iEEG power, the remaining channels were grouped into blocks, and re-referenced using a common average reference (CAR) filter within each block:

$$X{\left(T\right)}_{n}^{CAR}=X{\left(t\right)}_{n}-\frac{1}{N}\sum_{k=1}^{N}X{\left(t\right)}_{k}$$

where *X*(*t*)_*n*_ represents the raw iEEG power on the nth channel, and $X{\left(t\right)}_{n}^{CAR}$ represents the CAR-filtered iEEG power on the nth channel out of N recorded channels in a block after excluding bad channels. For Patients 1–4 (subdural grids/strips), separate CAR blocks were used for all the standard electrodes and all the high-density electrodes in each patient; for Patient 5 (SEEG depths), separate CAR blocks were used for each individual shank of depth electrodes.

### ESM Analysis

All patients underwent functional mapping with electrocortical stimulation of motor and language cortex following routine clinical procedures (Lesser et al., 1987; Sinai et al., 2005). ESM was performed in 2- to 3-h blocks over 1–2 days. Electrode pairs were stimulated using either a GRASS S-12 Biphasic Stimulator (Grass-Telefactor/Astro-Med, Inc., West Warwick, RI, USA) or a Nihon Kohden MS-120BK Cortical Stimulator (Nihon Kohden America, Irvine, CA, USA). Intracranial EEG was continuously monitored for after discharges and seizures. Two- to five seconds trains of 50 Hz, 0.3-ms, alternating polarity square-wave pulses were delivered in 0.5-mA increments, titrated from 1-mA up to a maximum of 12-mA (typically between 7 and 12-mA), or the highest amperage that did not produce after discharges at a given electrode pair, maximizing currents at each cortical site regardless of adjacent after discharge thresholds (Lesser et al., 1984; Pouratian et al., 2004). If ESM interfered with voluntary movement or produced involuntary movement or unpleasant sensations in one or more body parts, ESM mapping of language function was not performed for that electrode pair. Language location was determined as follows: electrode pairs were considered ESM positive (ESM+) for language if stimulation resulted in absent or delayed responses, paraphasic errors, and/or incorrect responses not followed by after discharges during at least two trials at the same electrode pair, if these errors were also not present during baseline testing (Sinai et al., 2005). Otherwise, electrode pairs were defined as ESM negative (ESM−) for language, but not for hesitation, interruption or incorrect response.

### High Gamma Response Analysis

The CAR-filtered iEEG signal was analyzed for the duration of the task in 128 ms epochs of data with 112 ms overlap. The Fast Fourier transform (FFT) was computed on each window, and the resulting coefficients were then multiplied by a modified flat-top Gaussian window with cutoff between 70 and 150 Hz, with notch filters applied to 60 Hz and 120 Hz for line noise elimination. The bandpass-filtered spectrum was converted to high gamma power by zeroing the negative frequency components, doubling the positive frequency components, computing the inverse FFT, and taking the magnitude of the result (i.e., the Hilbert transform) (Bruns, 2004; Canolty et al., 2007). The resulting high gamma power was then log transformed to approximate a normal distribution and decimated to a temporal resolution of 16 ms using a moving average filter. More details of spectral feature extraction methods can be found in Wang et al. (2016). A site was marked STFM positive (STFM+) if it exhibited a significant task-related high gamma power increase, and STFM negative (STFM−) if there were no significant high gamma power increase.

### Statistical Analysis

The baseline window used for each task was defined as a period from 1,000 ms before stimulus onset to 200 ms before stimulus onset, and a baseline distribution was formed per channel from the pooled high gamma power values during this period. A two-way *t*-test was performed between the distribution for each time-channel bin and that channel’s baseline distribution. The resulting *p-values* were corrected for multiple comparisons using the Benjamini-Hochberg (BH) procedure, controlling the false discovery rate (FDR) at 0.05 (Benjamini and Hochberg, 1995; see Wang et al., 2016; Milsap et al., 2019 for more details).

### Web-based Online Functional Brain Mapping (WebFM)

We used WebFM, an in-house designed software system, to perform online STFMs and CCEPs in a web-browser window, using local processing at the bedside, and to synchronize the results to a centrally hosted repository (Milsap et al., 2019). The system leverages the real-time experimental control and signal analysis capabilities of BCI2000 (Schalk et al., 2004), a standardized software platform for brain-computer interface (BCI) research used by over 400 labs over the past 15 years that can interface with a wide variety of commercial EEG amplifiers. WebFM integrates with BCI2000 in a browser-based user interface for immediately displaying spatial-temporal maps of functional activation (and now CCEPs) during the course of testing at the patient’s bedside. This allows both researchers and clinicians to identify any technical problems during testing, ensure valid results, and to decide whether additional testing is needed.

### Cortico-Cortical Evoked Potentials (CCEPs)

The methods used to elicit and analyze CCEPs is described in detail elsewhere (Matsumoto et al., 2004, 2007). Direct electrical stimulation was applied in a bipolar manner to a pair of adjacently placed subdural electrodes using a constant-current stimulator (Grass S12 stimulator, AstroMed, Inc., West Warwick, RI, USA; or Cerestim R96, Blackrock Microsystems, LLC, Salt Lake City, UT, USA). Single-pulse electrical stimuli (SPES, biphasic wave pulse: 0.3-ms duration) were delivered at jittered interstimulus intervals of 2–5 s (Patient 1: 2–2.5 s; Patient 2–4: 5 s; Patient 5: 2.5 s). The stimulation was monitored by physicians (NC and JK) experienced in iEEG interpretation and electrocortical stimulation mapping. One set of trials for each stimulation site comprised 50–167 stimuli (Patient 1: 51–167; Patient 2: 60; Patient 3–5: 50), depending on clinical circumstances and the time available for testing. During the recording, we asked the patients to recline comfortably awake on the bed and to continue their usual activities. We titrated stimulation intensity in increments of 0.5–1 mA, making sure that no afterdischarges were induced, up to a maximum of 5 mA for electrode arrays with 0.5 cm spacing or 10 mA for arrays with 1.0 cm spacing. More current was used for electrodes with larger surface area and interelectrode distances to achieve similar current densities. Because stimulation of sensorimotor or visual cortices can sometimes evoke symptoms at low intensities (i.e., movement or subjective sensory sensation of a part of the body, and phosphenes), we sometimes used 5 mA even for arrays with 1.0 cm spacing and confirmed that stimulation produced no symptom. At the maximum current for each stimulation, the N1 potential in the average CCEP response of each electrode was identified within 10–50 ms post-stimulus, thought to represent a direct excitatory connection from stimulation to response site (Matsumoto et al., 2004, 2007). Observed N1 latencies in our dataset had a mean of 9.63 ms and standard deviation of 8.94 ms. The magnitude of the N1 potential was normalized using the pre-stimulus baseline (−500 ms to −10 ms) to obtain a *z*-score that quantifies that electrode’s effective connectivity.

### Logistic Regression Model

We trained a logistic regression model to predict ESM results at individual electrode pairs using a linear combination of measures derived from STFM alone, or STFM and CCEPs (see Equations 1, 2 and 3 below). In order to validate the reliability of the model, we used a 5-fold cross-validation to protect against overfitting by partitioning the dataset into five folds and estimating accuracy in each fold.

Equation 1: ESM = $\alpha_{1}\frac{\sum_{Stim}(HG_{All\;Time})}{2}+\alpha_{2}$, where *α*_1_ and *α*_2_ are model coefficients.

Equation 2: ESM = $\beta_{1}\frac{\sum_{Stim}(HG_{PCA})}{2}+\beta_{2}$, where *β*_1_ and *β*_2_ are model coefficients.

Equation 3: ESM = $\gamma_{1}\frac{\sum_{Stim}(HG_{PCA})}{2}+\gamma_{2}\frac{\sum_{Stim}(Centrality_{HG})}{2}+\gamma_{3}\frac{\sum_{grid}(CCEPs\times HG_{PCA})}{\#Electrodes}+\gamma_{4}$, where *γ*_1_, *γ*_2_, *γ*_3_ and *γ*_4_ are model coefficients.

As a first approximation of the degree of activation (Equation 1) we integrated the magnitude of HG power increases over the duration of the task (*HG*_All time_). For a more temporally specific measure of activation (Equation 2), we integrated the magnitude of HG power increases within windows defined by principal component analysis of the HG time series (*HG*_PCA_) (Collard et al., 2016). To estimate the importance of STFM+ sites to overall network activation (Equation 3), we computed Pearson’s linear correlation coefficients between the temporal envelopes of high gamma power increases at all STFM+ sites (Collard et al., 2016), and then we computed the PageRank centrality of each STFM+ site (*Centrality*_HG_). To estimate the effective connectivity of ESM stimulation sites with the overall network of task-activated cortical sites, we computed the sum, across all sites with CCEPs, of the product of the (*HG*_PCA_) at each site and the *z-score* of the CCEP elicited at that site by SPES at the ESM stimulation site. To account for potential ESM effects on an even broader network, including sites without significant (*HG*_PCA_), we also computed the sum, across all sites with CCEPs, of the product of the (*HG*_PCA_) at each site and the total number of significant CCEPs elicited by SPES at the ESM stimulation site.

## Results

**Figure 1:**
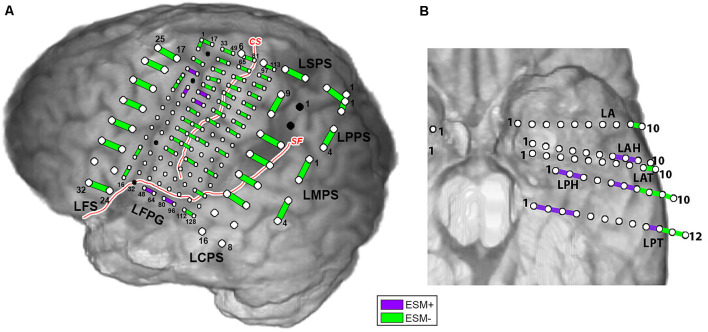
Electrocortical stimulation mapping (ESM) results for Patient 2 **(A)** and Patient 5 **(B)**. Purple bars indicate electrode pairs that were positive during any language task (ESM+); green bars indicate those that were negative during all language tasks (ESM−). Red curves with white stroke represent anatomical landmarks—central sulcus (CS) and sylvian fissure (SF).

Examples of electrocortical stimulation mapping (ESM) results, for Patient 2 (subdural grids/strips) and Patient 5 (SEEG depth electrodes), are illustrated in Figure 1. Electrode pairs positive for language are shown in purple, and the ones negative for language are shown in green. ESM positive pairs are entered as value 1, and negative pairs entered as value 0 as the observed responses in the prediction model (for Equations 1, 2 and 3). For Patient 2, ESM+ pairs were located around left superior temporal gyrus (LFPG48-64, LFPG80-96) as well as left superior precentral gyrus (LFPG38-54, LFPG39-55) and premotor area (LFPG4-20, LFPG22-23). For Patient 5, ESM+ pairs were located along left hippocampus (LPH1-2, LPT1-2, 2-3), left fusiform gyrus (LPH2-3, LPT3-4), left superior temporal gyrus (LAH7-8, LAH8-9, LPH6-7), and left middle temporal gyrus (LPH5-6, LPT 9-10).

**Figure 2:**
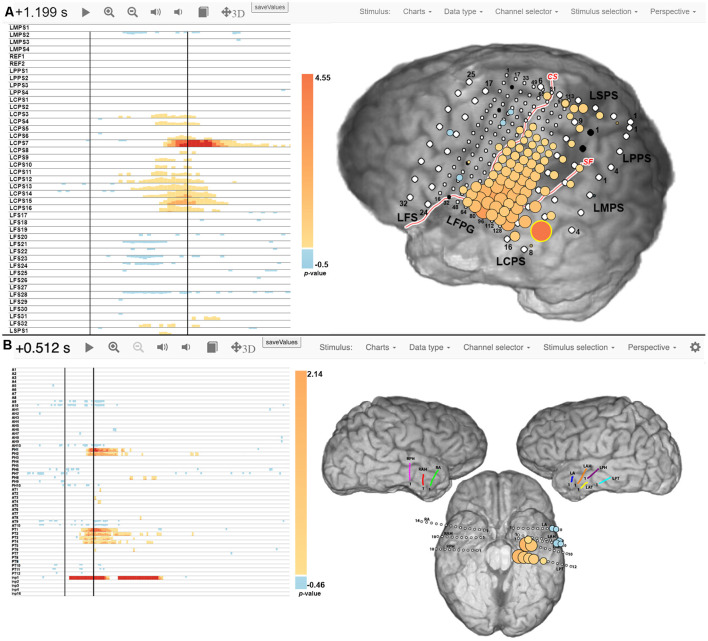
Spatial-temporal functional mapping (STFM) results for Patient 2 during a word reading task **(A)** and Patient 5 during a picture naming task **(B)**. The raster plot on the left shows trial-averaged STFM results in a time by channels manner, and the brain map on the right shows STFM results on an anatomical illustration at a specific time stamp. More details can be found at Wang et al. (2016) and Milsap et al. (2019).

Figure 2 shows examples of spatial-temporal function mapping (STFM) results, for Patient 2 during a word reading task, and Patient 5 during a picture naming task. For each map, a raster plot on the left displays the magnitude of event-related changes in the high gamma analytic power at each time point after stimulus onset, as compared to the pre-stimulus baseline. The magnitudes are thresholded for significance (*p* < 0.05) using False Discovery Rate (FDR) correction for each channel in the channel raster. A brain map is displayed on the right to show the locations and relative magnitudes of activations either integrated over the entire post-stimulus interval or at any user-selectable time point in the channel raster (*t* = 1.20 s and *t* = 0.51 s post-stimulus onset shown in Figure 2, respectively). The magnitude of the high gamma power at a particular electrode and time is represented by the size and color of disks overlaid on iEEG electrode locations in a two-dimensional snapshot of the three-dimensional brain reconstruction. For Patient 2 during word reading, STFM+ electrodes were located around superior temporal gyrus, as well as pre- and post-central gyri. For Patient 5 during picture naming, STFM+ electrodes were located along left hippocampus, left fusiform gyrus, left superior temporal gyrus, and left middle temporal gyrus. STFM results for all patients in this article are available at http://webfm.io.

Note that ESM+ and STFM+ were sometimes observed outside of classical language areas. In some cases, these sites were in sensorimotor cortex and likely corresponded to preparation and execution of spoken responses. In other cases, they may have been due to reorganization in response to a prior surgical resection in the parietal cortex.

**Figure 3:**
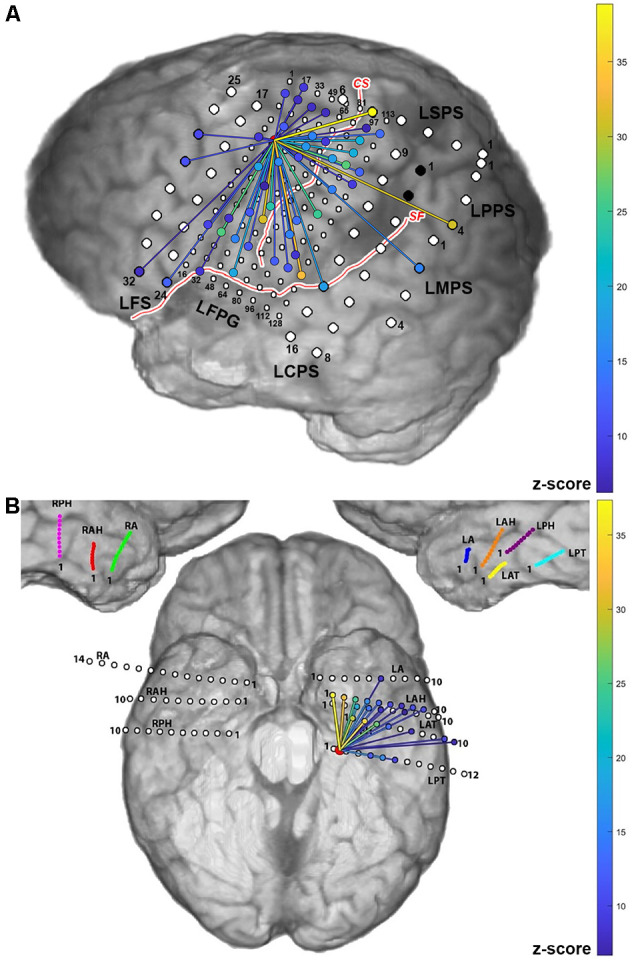
Cortico-cortical evoked potential (CCEP) results for Patients 2 **(A)** and 5 **(B)** when stimulating individual electrode pairs (red circle between stimulated electrodes. The normalized response amplitude of the average CCEP observed at each electrode, defined as that electrode’s *z*-score, is used to quantify the effective connectivity between the stimulation and response site. Responses with a *z*-score greater than 6 were considered significant and are represented here as lines from the stimulation site (denoted in red) to the sites with significant CCEPs, colored according to the magnitude of the *z-score* observed at that electrode (color scale shown on right).

Examples of cortico-cortical evoked potential (CCEPs) results for one stimulation pair (LFPG21-LFPG22) in Patient 2, and one pair (LPT1–LPT2) in Patient 5, are shown in Figure 3. The normalized response amplitudes of the average CCEPs observed at each electrode, defined as that electrode’s *z*-score relative to the pre-stimulus baseline, was used to quantify the effective connectivity between the stimulation and response site. Responses with a *z-score* greater than 6 were considered significant, as a previous study has shown this threshold to optimize sensitivity and specificity in identification of CCEP responses (Keller et al., 2011). Significant responses are represented here as lines from the stimulation site to the sites with significant CCEPs.

Based on Equations 1, 2 and 3 from “Materials and Methods” section: Logistic Regression Model, we trained our classifier using generalized linear model. The model accuracy for Patients 2 and 5 is listed on Table 2 as examples. The average accuracy of the classifier was 76.5% and 52.9% for patients 2 and 5, respectively, using high gamma power integrated over the duration of the task (Equation 1), 76.5% and 58.8% using high gamma power within windows defined by PCA (Equation 2), and 82.4% and 88.2% using high gamma power and centrality/CCEP results combined, respectively (Equation 3).

**Table 2:** Model accuracy for Patients 2 (word reading) and 5 (picture naming).

	Accuracy (%)	STFM+/ESM+	STFM−/ESM+	STFM−/ESM−	STFM+/ESM−	Sensitivity (%)	Specificity (%)	AUC	*p*-value	Equation
Patient 2
HG all duration	76.5%	8	3	5	1	72.7%	83.3%	0.79	0.0465	(1)
HG PCA selected duartion	76.5%	8	3	5	1	72.7%	83.3%	0.7	0.0439	(2)
HG scaled by centrality	82.4%	10	1	4	2	90.9%	66.7%	0.83	0.134	(3) γ_3_ = 0
HG + CCEPs *z*-score	82.4%	9	2	5	1	81.8%	83.3%	0.83	0.103	(3) CCEPs = *z*-score
HG + CCEPs edges	82.4%	9	2	5	1	81.8%	83.3%	0.86	0.0747	(3) CCEPs = Edges
Patient 5
HG all duration	52.9%	9	1	0	7	90.0%	0.0%	0.31	0.881	(1)
HG PCA selected duartion	58.8%	8	2	2	5	80.0%	28.6%	0.39	0.952	(2)
HG scaled by centrality	82.4%	8	2	6	1	80.0%	85.7%	0.73	0.947	(3) γ_3_ = 0
HG scaled by CCEPs *z*-score	82.4%	8	2	6	1	80.0%	85.7%	0.8	0.0673	(3) CCEPs = *z*-score
HG scaled by CCEPs edges	88.2%	9	1	6	1	90.0%	85.7%	0.81	0.0462	(3) CCEPs = Edges

*Accuracy (%), areas under ROC curve (AUC), and p-values were calculated using Classification Learner from Matlab. Sensitivity was calculated using STFM+ESM+ and STFM−ESM+ electrode numbers, and specificity using STFM−ESM− and STFM+ESM− electrodes. Results for Equation 1 are listed on the first row of each subtable; results for Equation 2 are listed on the second row; results for Equation 3 are listed in several steps on the rest of the rows*.

**Figure 4:**
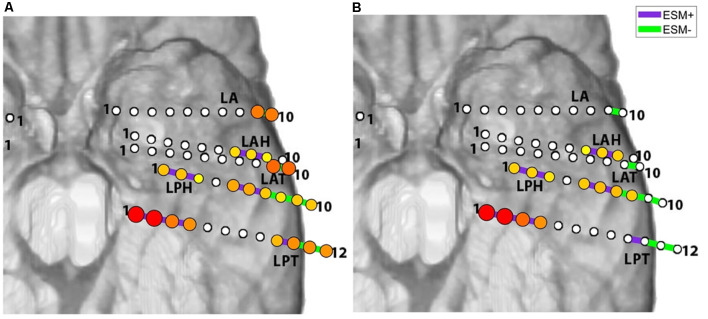
ESM vs. its predictions by different models, for picture naming for Patient 5. Model in **(A)** is based on task-related STFM alone. Model in **(B)** uses STFM and CCEPs to estimate importance of sites to overall network dynamics and connectivity of ESM+ sites (purple bars) to other sites of importance to network dynamics. Note that ESM− sites have fewer HGM+ sites under the model on the right.

Figure 4 illustrates how ESM compares to its prediction by two different models: one in which only STFM is used (left) and one in which both STFM and CCEPs results are used (right). These illustrate how the correspondence between passive iEEG maps and ESM results is greater when the effective connectivity of ESM stimulation sites with a larger network of task- activated sites is taken into consideration.

As shown in Table 3, the average accuracy and area under the receiver operating characteristic curve (AUC) of the classifier was 71.8% and 0.43 respectively, using high gamma power integrated over the duration of the task (Equation 1), 74.4% and 0.55 respectively, using high gamma power within windows defined by PCA (Equation 2), and 87.7% and 0.83 using high gamma power and centrality/CCEP results combined (Equation 3). We then performed a two-sample *t*-test between results using Equations 2 and 3, and the *p-values* for differences between accuracy and AUC under the two models were 0.022 and 0.046 respectively, meaning that both accuracy and AUC using Equation 3 were statistically larger than those using Equation 2 (*p* < 0.05).

**Table 3:** Summary of classification accuracy and area under curve using different classification models.

Patient	Accuracy (%)	AUC	Accuracy (%)	AUC	Accuracy (%)	AUC
	Model 1	Model 1	Model 2	Model 2	Model 3	Model 3
1	76.5	0.70	76.5	0.83	82.4	0.86
2	75.0	0.19	75.0	0.31	83.3	0.56
3	71.4	0.25	78.6	0.48	92.9	0.92
4	83.3	0.72	83.3	0.72	91.7	1.00
5	52.9	0.31	58.8	0.39	88.2	0.81
Averaged	71.8	0.43	74.4*	0.55**	87.7*	0.83**

The two numbers with * (accuracy) and two numbers with ** (AUC) indicates that the two-sample *t*-test rejects the null hypothesis that the two numbers are equal at the 5% significance level.

## Discussion

Results from the five patients performing a variety of language tasks demonstrated that combining the results of iEEG STFM and CCEPs improved the accuracy of predicting ESM results for language functional mapping during intracranial monitoring for epilepsy surgery.

ESM is still the gold-standard for localizing eloquent cortex, but when compared to ground-truth patient outcomes, unpredicted resective deficits can and do still occur (Sinai et al., 2005; Hamberger, 2007; Asano et al., 2009; Cervenka et al., 2011, 2013; Kojima et al., 2012; Sakpichaisakul et al., 2020). Passive iEEG mapping has been investigated as a replacement for ESM, but to date, its combined sensitivity and specificity relative to ESM have been suboptimal, especially for language mapping (Bauer et al., 2013). Both methods have potential strengths and limitations. ESM reversibly simulates the behavioral effect of a lesion at the site of stimulation, but its effects, particularly for long trains, may not be confined to the stimulation site, potentially eliciting action potentials in fibers of passage and interfering with function at distant sites.

**Figure 5:**
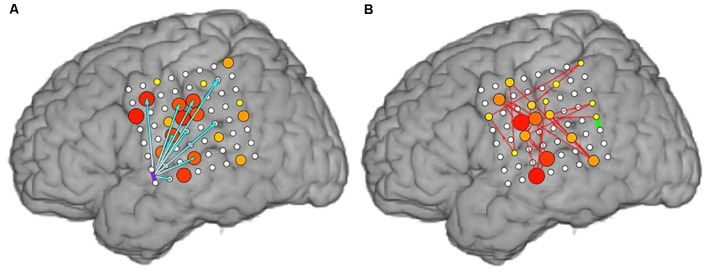
**(A)** STFM−/ESM+ (false negative) sites are hypothesized to result from distant effects of ESM (purple bar) on a broader network of STFM+ sites (cyan arrows). These effects may depend on the importance of STFM+ sites to task performance, estimated by activation magnitude and/or network centrality (color and size of electrodes). **(B)** Conversely, STFM+/ESM− (false positive) sites (green bar) are hypothesized to have low importance in overall task-related network function because of other STFM+ sites of equal or greater magnitude of activation and/or centrality (color and size of electrodes) to network dynamics (red arrows).

Indeed, evidence from a variety of sources has called into question the assumption that the effects of ESM are restricted to tissue underneath each stimulating electrode. First, a large number of studies have shown that brief single pulses of cortical stimulation at intensities comparable to ESM can elicit evoked cortical potentials (Matsumoto et al., 2004), as well as changes in high gamma power (and thus population firing rates) (Usami et al., 2015, 2019). Cortico-cortical evoked potentials (CCEPs) have been increasingly used to map the effective connectivity between the cortical components of language networks and motor networks (Matsumoto et al., 2007, 2017; Tamura et al., 2016). Although single-pulse electrical stimulation (SPES) is too brief to disrupt cortical function locally or at distant sites, the CCEPs elicited by SPES provide direct evidence for distant electrophysiological effects from ESM. ESM typically employs 50 Hz trains of stimuli at the same or greater intensity as SPES for several seconds, and can thus recruit a far greater neuronal population than SPES, both locally and distantly. However, the massive stimulus artifact created by ESM usually obscures any potentials elicited either locally or at distant sites. Yet, investigators have observed trains of intra-stimulation potentials locked to ESM stimulation trains at cortical sites several centimeters away from the site of ESM (Karakis et al., 2015). In one such report, stimulation in sub-temporal cortex elicited discharges in left posterior superior temporal gyrus that were associated with language impairment (Ishitobi et al., 2000). Based on these findings we hypothesize that at least some false negative STFM sites are due to the effects of ESM on a broader network of STFM+ sites (see left panel of Figure 5 for illustration).

A longstanding concern for mapping techniques that depend on functional activation (e.g., PET, fMRI) is that sites may be recruited without being critical to task performance. Likewise, STFM+ sites may sometimes be ESM−. We hypothesize that some of these STFM false positives occur because the site plays a relatively minor role in task performance. This could be because there are other sites with equal or greater activation and/or because the site’s centrality to network dynamics is low (see right panel of Figure 5 for illustration).

Previously, our team had taken an admittedly coarse approach to validation of STFM by testing its ability to independently identify functional cortex defined by regions of interest (ROIs) drawn from the literature. Using this approach, we made a preliminary case for arguing that iEEG STFM outperforms ESM (Wang et al., 2016). In this article, we provide evidence that the correspondence between STFM and ESM results is significantly greater when the effective connectivity between ESM stimulation sites and a larger network of task-activated sites is taken into consideration. Ultimately, however, STFM will need to show superior performance in predicting post-operative deficits to truly replace ESM (Arya et al., 2019b). In addition to being an independent standard, predicting post-operative deficits is the ultimate goal of epilepsy surgery planning.

The use of post-operative deficits as a gold standard for validation of ESM and STFM is certainly not without its own challenges. Aggregating databases of post-operative deficits and their relationships with functional mapping results is difficult since putative eloquent cortex identified by either technique is usually spared. This can lead to an underrepresentation of resected ESM+ sites. The net effect this sampling bias can potentially overestimate STFM’s relative sensitivity and ESM’s relative specificity. This could potentially be overcome by including enough patients in the database such that these factors are balanced appropriately. Beyond the issue of sampling bias, however, is the complication that deficits are not truly binary: they may be incomplete or vary in duration (e.g., deficits may be chronic or resolve several months after surgery). Segmenting the database into sub-categories could potentially highlight a role for STFM but would further increase the size requirements of the cohort.

## Conclusions

Our study represents a preliminary attempt to reconcile inconsistencies between the results of STFM and ESM that we and other groups have observed. Agreement between these methods has not been as good for language mapping as it has been for motor mapping. Here, we hypothesized that incongruities between ESM and STFM of language function may arise due to propagation of the effects of ESM to cortical areas with strong effective connectivity with the site of stimulation. To estimate the effective connectivity of stimulation sites with a broader network of task-activated cortical sites, we measured CCEPs elicited across all recording sites by single-pulse electrical stimulation at sites where ESM was performed at other times. With the combination of STFM as well as CCEP results, we were able to train a logistic regression model to significantly better predict ESM results at individual electrode pairs. Our findings suggest that the correspondence between STFM and ESM results is greater when the effective connectivity of ESM stimulation sites with a larger network of task-activated sites is taken into consideration. This provides preliminary support for the hypothesis that ESM can have distant effects on a wider network of cortical language sites and suggests that cortical network mapping with CCEPs may help resolve the observed discrepancies between ESM and STFM results. Because these findings are based on a relatively small number of subjects to date, we believe that studies with more patients are needed, including studies of post-operative outcomes, to understand the relative strengths and weaknesses of STFM and ESM, and their comparative utilities for mapping language cortex in advance of surgical resections.

## Data Availability Statement

The raw data supporting the conclusions of this article will be made available by the authors, without undue reservation.

## Ethics Statement

The studies involving human participants were reviewed and approved by the Johns Hopkins Medicine Institutional Review Boards. The patients/participants provided their written informed consent to participate in this study.

## Author Contributions

YW and NC designed the study. YW, MH, CC and JK conducted the experiments. YW and MH analyzed the data. YW, CC and MH generated figures in the article. YW wrote the article with input from all authors. AK and NC supervised the project. All authors contributed to the article and approved the submitted version.

## Conflict of Interest

The authors declare that the research was conducted in the absence of any commercial or financial relationships that could be construed as a potential conflict of interest.
